# Effects of Lateral and Terminal Chains of X-Shaped Bolapolyphiles with Oligo(phenylene ethynylene) Cores on Self-Assembly Behavior. Part 2: Domain Formation by Self-Assembly in Lipid Bilayer Membranes

**DOI:** 10.3390/polym9100476

**Published:** 2017-09-29

**Authors:** Stefan Werner, Jan Ebenhan, Marco Poppe, Silvio Poppe, Helgard Ebert, Carsten Tschierske, Kirsten Bacia

**Affiliations:** 1 Department of Chemistry, Martin Luther University Halle-Wittenberg, Kurt-Mothes-Str. 3, 06120 Halle, Germany; stefan.werner@chemie.uni-halle.de (S.W.); jan.ebenhan@chemie.uni-halle.de (J.E.); 2 Department of Chemistry, Martin Luther University Halle-Wittenberg, Kurt-Mothes-Str. 2, 06120 Halle, Germany; marco.poppe@googlemail.com (M.P.); silviopoppe@googlemail.com (S.P.); Helgard.Ebert@gmx.de (H.E.); carsten.tschierske@chemie.uni-halle.de (C.T.)

**Keywords:** self-organization, self-assembly, amphiphiles, polyphiles, bolaamphiphiles, confocal fluorescence microscopy, membrane organization, phase separation, giant unilamellar vesicles, umbrella model

## Abstract

Supramolecular self-assembly of membrane constituents within a phospholipid bilayer creates complex functional platforms in biological cells that operate in intracellular signaling, trafficking and membrane remodeling. Synthetic polyphilic compounds of macromolecular or small size can be incorporated into artificial phospholipid bilayers. Featuring three or four moieties of different philicities, they reach beyond ordinary amphiphilicity and open up avenues to new functions and interaction concepts. Here, we have incorporated a series of X-shaped bolapolyphiles into DPPC (1,2-dipalmitoyl-*sn*-glycero-3-phosphocholine) bilayers of giant unilamellar vesicles. The bolapolyphiles consist of a rod-like oligo(phenylene ethynylene) (OPE) core, hydrophilic glycerol-based headgroups with or without oligo(ethylene oxide) expansions at both ends and two lateral alkyl chains attached near the center of the OPE core. In the absence of DPPC and water, the compounds showed thermotropic liquid-crystalline behavior with a transition between polyphilic and amphiphilic assembly (see part 1 in this issue). In DPPC membranes, various trends in the domain morphologies were observed upon structure variations, which entailed branched alkyl chains of various sizes, alkyl chain semiperfluorination and size expansion of the headgroups. Observed effects on domain morphology are interpreted in the context of the bulk behavior (part 1) and of a model that was previously developed based on spectroscopic and physicochemical data.

## 1. Introduction

Supramolecular self-assembly generates complex structures both in purely synthetic and in biological systems. A number of functional entities in biological cells are dynamically created by supramolecular polymerization of biomolecules. Protein assemblies include for instance cytoskeletal filaments formed by the linear polymerization of actin [1] and by the tubular polymerization of tubulin [2] and cage-like protein lattices with multifold symmetries serving intracellular vesicle formation [3] and virus assembly [4], to name only a few prominent examples. Assemblies with a plethora of symmetries are encountered [5]. Extended protein complexes and molecular machines are formed and held together by non-covalent interactions, including the hydrophobic effect, aromatic interactions, polar interactions, hydrogen-bonding and van-der-Waals interactions. An interplay of rigid and flexible moieties is essential for the function of such protein complexes.

There is continually-growing evidence that the non-covalent supramolecular self-assembly of membrane constituents within the plane of biological membranes is functionally of similar importance as complex formation in an aqueous environment. Controlled lateral aggregation of selected lipid and protein constituents leads to the formation of microdomains that serve as platforms for cellular signaling as well for vesicle budding and trafficking [6]. However, there are particular challenges to studying complex self-assembly phenomena in lipid bilayers compared to studies of self-assembly in the aqueous cytosol. One challenge is that lipids cannot be targeted directly by genetic approaches for testing structural variations as it is feasible for proteins. Another challenge is presented by the sheet-like topology of the lipid bilayer membrane with its hydrophobic alkyl chain core sandwiched between two opposing hydrophilic headgroup regions. This amphiphilic bilayer imposes a restraint on the orientation of amphiphilic membrane constituents, but it is also adaptive. For example, a lipid bilayer responds to a mismatch in hydrophobic length between lipids and membrane proteins [7]. The bilayer is also able to accommodate a combination of macromolecular amphiphilic block-copolymers and small amphiphilic lipids [8]. Nonetheless, hydrophobic mismatch is a driving force for lateral segregation, leading to the formation of lipid-enriched and macromolecule-enriched membrane domains, respectively [8,9].

Artificial vesicles constitute widely-used model systems for mimicking cellular membranes and for studying the fundamental principles of the underlying supramolecular assembly, because their composition can be tuned at will. By virtue of their free-standing bilayers, unilamellar vesicles are particularly suited for studying domain formation processes, where a solid membrane support constitutes a perturbation that influences aggregation processes [10]. Owing to their size, giant unilamellar vesicles (GUVs) are especially useful for visualizing micron-scale assembly processes by confocal fluorescence microscopy.

In this report, we investigate the assembly of a series of specifically designed, synthetic polyphilic molecules within the plane of a lipid bilayer to further elucidate the relationship between the molecular structure and resulting supramolecular assembly. In previous reports [11,12,13], we focused on the X-shaped bolapolyphile **B12**, which consists of three key elements, each featuring a particular philicity: a 3.1 nm long oligo(phenylene ethynylene) (OPE) core, which forms a π-conjugated, hydrophobic and stiff rod; two terminal glycerol groups, which are polar, hydrophilic and capable of hydrogen-bonding; and two saturated C_12_ chains, connected at the central phenyl ring on opposite sides of the hydrophobic rod, which are lipophilic and flexible. In the presence of excess water, bilayers are formed by this synthetic bola-type molecule in mixtures with the lipid DPPC (1,2-dipalmitoyl-*sn*-glycero-3-phosphocholine).

**Scheme 1:**
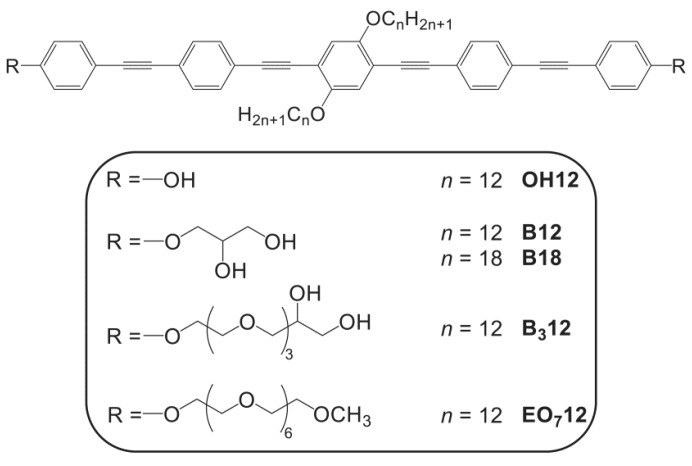
Chemical structures of bolapolyphile compounds previously studied in DPPC lipid bilayers [12] with names according to the nomenclature in part 1 [17], where applicable.

The **B12** bolapolyphile serves as a model synthetic compound to elucidate principles of supramolecular structure formation in membranes. With its rigid hydrophobic moiety of defined hydrophobic length, akin to the rigid α-helical transmembrane proteins, its headgroup with hydrogen-bonding capability and the lateral alkyl chains, **B12** encompasses more philicities than the standard amphiphilic head-and-tail configuration of lipids or the head-tail-head configuration of bolalipids [14,15,16]. We have previously explored the molecular interactions and the structure formation enabled by these philicities in **B12**:DPPC bilayers by calorimetry (DSC), X-ray diffraction (XRD) and nuclear magnetic resonance (NMR) spectroscopy, leading to a model of supramolecular **B12** assembly [11,12]. Based on these data, it appears that **B12** molecules pack into π-π stacked filaments with additional stabilization from hydrogen-bonding between the headgroups, in analogy to the stacking of **B12** and related compounds in the absence of water [17], while having a roughly transverse orientation in the membrane. The hydrophilic glycerol headgroups of **B12** interact with the phospholipid headgroups in either leaflet, while the lateral alkyl chains interact with the chains of the phospholipid molecules in the hydrophobic membrane core. Based on the observation of snowflake-like hexagonal symmetry on the large micrometer-scale, we hypothesized that, on the nanoscale, the **B12** filaments surround the phospholipids, whose alkyl chains mix with the **B12** lateral chains, in a hexagonal (honeycomb) pattern. We also sampled selected chemical variations by replacing the C_12_ alkyl chains by C_18_ alkyl chains (**B18**), by substituting the glycerols with simple hydroxy groups, by extending the headgroup with a tri(ethylene oxide) chain in between the OPE core and glycerols (**B_3_12**, formerly called **D12/3**) and by extending the headgroup with methoxy-terminated hepta(ethylene oxide) (**EO_7_12**) (see Scheme 1 for structures). The large scale snowflake-like hexagonal symmetry was preserved for **B18** and for the compound with simple hydroxy headgroups (**OH12**), but not for the two compounds with expanded polar groups (**B_3_12** and **EO_7_12**) [12].

**Scheme 2:**
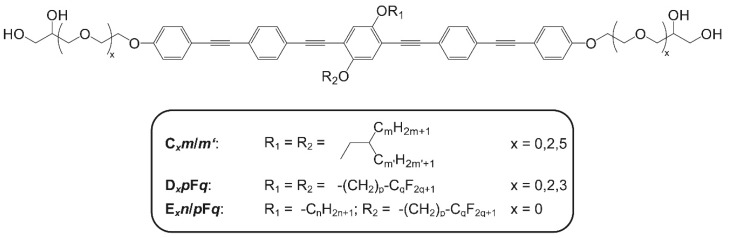
Bolapolyphile compounds investigated in DPPC lipid bilayers in this study (see part 1 [17] for synthesis and bulk behavior). The index x is omitted in the case of x = 0.

Here, we have considerably extended the investigations into the large-scale morphological effects of molecular scale chemical variations by nineteen more molecular structures. The first series of compounds comprises symmetrically-branched lateral alkyl chains (**C*m/m’*** with *m* = *m*’: **C2/2, C4/4, C8/8**, **C12/12**, **C13/13**, **C16/16**, **C18/18**, **C22/22**) and non-symmetrically-branched lateral alkyl chains (**C*m/m’*** with *m* ≠ *m*’: **C16/4**, **C16/14**, **C18/12**, see Scheme 2 for structures). These were compared to previously studied compounds with unbranched chains (**B12**, **B18**). To facilitate comparison between side chain volumes in the B and C series, the total number of carbon atoms per lateral residue is provided in curly brackets behind the compound name. For the B series, the number of carbon atoms per lateral chain equals n, for example, **B12** {12}. For the C series, the total number of carbon atoms per lateral chain equals 2 + *m* + *m’*, for example **C16/14** {32}.

Secondly, compounds containing semiperfluorination as a fourth philicity at only one (**E16/4F10**, **E18/4F6** and **E18/4F10**) or both (**D4F6**) of the unbranched lateral chains were tested in the same experimental system.

Thirdly, relating to the previously studied compound **B_3_12**, which features an expanded (ethylene oxide)_3_ headgroup, new compounds with expanded headgroups and branched chains (**C_3_12/12**, **C_5_12/12**) as well as compounds with expanded headgroups and unbranched semiperfluorinated chains (**D_2_4F6**, **D_3_4F6**) were investigated in the lipid bilayer system.

The natural glycerophospholipid DPPC, which has a main transition temperature of 41 °C [18] has been widely used in investigations of phase behavior and structure formation in fully-hydrated lipid bilayers. At room temperature, DPPC forms a solid-ordered (*s_o_*) gel phase, where the lipid chains are mostly in an ordered all-*trans*-configuration, the lipids are tilted at an angle of approximately 30° and the headgroups fill the 2D-space by hexagonal packing (*L_β’_* phase) [19].

To explore phase behavior and domain formation, many studies have combined DPPC with lipids of lower melting points and/or with planar, order-inducing sterols such as cholesterol [20]. Low-*T_m_* lipids assemble into a liquid-disordered (*l_d_*) phase at room temperature, meaning a fluid phase with highly mobile chains (*L_α_*). Although, due to its small hydroxy-headgroup, cholesterol is not able to form a bilayer by itself, it packs tightly with saturated chain lipids such as DPPC in bilayers, leading to the formation of a liquid-ordered (*l_o_*) phase [21]. We therefore chose DPPC as a model lipid in combination with the newly synthesized bolapolyphiles for exploring lateral domain formation.

## 2. Materials and Methods

1,2-Dipalmitoyl-*sn*-glycero-3-phosphatidylcholine (DPPC) and the red fluorescent lipid marker Rh-DPPE (1,2-dipalmitoyl-*sn*-glycero-3-phosphoethanolamine-*N*-(lissamine rhodamine B sulfonyl) (ammonium salt)) were purchased from Avanti Polar Lipids (Alabaster, AL, USA). All substances were used without further purification. Synthesis and characterization of the bulk self-assembly of most of the bolapolyphiles are described in part 1 [17]. The synthesis of the non-symmetrically branched compounds **C16/4, C16/14, C18/12** and the fluorinated compounds with long fluorinated segments (**En/4F10**) was conducted in analogy to the procedures given for related compounds [17,22,23]. Details of the synthesis and investigation of the bulk self-assembly of these compounds will be reported separately in forthcoming reports.

Giant unilamellar vesicles (GUVs) were produced by electroformation, following the same protocol as in [12]. Briefly, DPPC and a bolapolyphile were dissolved in chloroform at the desired molar ratio of 10:1 and 0.5 mol % Rh-PE was added. The solution was spread on a preheated indium-tin-oxide- (ITO) coated coverslip at 60 °C to evaporate the solvent. A perfusion chamber was assembled using two ITO-coverslips and a 2 mm spacer and filled with pure water. The conductive ITO coatings of the coverslips were connected to a function generator for electroformation (10 Hz sinusoidal, *V*_RMS_ = 1.3 V, 4 h). After disconnecting, the chamber was allowed to cool to room temperature and examined by confocal fluorescence microscopy on an inverted LSM710 microscope (Carl Zeiss, Jena, Germany) at 22 °C [12], using a 40 × 1.2 NA C-Apochromat objective. A 405 nm diode laser was used to excite the bolapolyphile, and fluorescence emission was collected from λ = 412 to 500 nm. The Rh-PE was excited by a 561 nm DPSS laser and emission collected from 566 to 681 nm. Emissions were separated by means of an internal grating. Photomultipliers served as detectors. Both color channels were recorded simultaneously.

In the figures, the bolapolyphile autofluorescence is displayed in green and the Rh-DPPE fluorescence is depicted in red pseudocolor. All figure panels show red and green overlays of maximum intensity projections, obtained from an axial series of confocal slices, with the axial range chosen to display a hemisphere of one GUV in the center of the panel. Other vesicles lie on top of each other or, in some instances, are only partially captured in the axial dimension, yielding an artefactual impression of a central hole. Small vesicles are prone to moving in between slice acquisitions. Lateral optical resolution is of the order of 200 nm. The appearance of concentric rings is a sampling artifact that depends on the axial increment between slices relative to the size of the confocal detection volume.

## 3. Results and Discussion

### 3.1. The GUV Model System

Here, we examine the supramolecular self-assembly of bolapolyphile compounds in free-standing lipid bilayers. A previously established model system consisting of GUVs prepared from the lipid DPPC and a bolapolyphile at a 10:1 molar ratio [11,12,13] was applied to each of the newly-synthesized bolapolyphile compounds from the three series described in the introduction. The first series included those with branched alkyl chains to investigate the role of side-chain space requirement, the second series included semiperfluorination at one or both lateral chains to study the effect of introducing a fourth philicity, and the third series contained oligo(ethylene oxide) (EO) linkers to explore the effect of expanded, more flexible headgroups.

**Figure 1:**
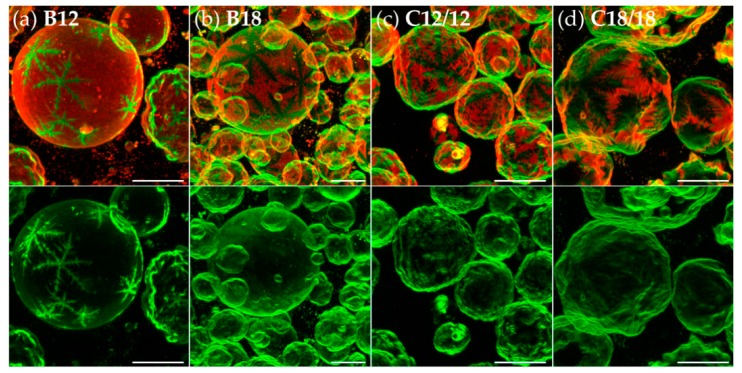
Dendritic six-fold symmetry stars formed with branched and unbranched alkyl chains. Confocal 3D projections of giant unilamellar vesicles (GUVs) from 1,2-dipalmitoyl-*sn*-glycero-3-phosphocholine (DPPC) and different bolapolyphiles ((**a**) **B12** {12}, (**b**) **B18** {18}, (**c**) **C12/12** {26} and (**d**) **C18/18** {38}), prepared at a molar ratio of 1:10. {*n*} denotes the number of carbon atoms per lateral chain, which is used as a measure for lateral chain volume. Images have been sorted from left to right according to increasing lateral chain volume. In the upper row, the autofluorescence of the bolapolyphiles is displayed in green and the fluorescence of the Rh-DPPE counterstain is shown in red. In the corresponding panels in the lower row, the bolapolyphile fluorescence is displayed separately. Confocal images of GUVs containing **B12** {12} and **B18** {18} have previously been published in [12]. Scale bars = 20 µm.

**Figure 2:**
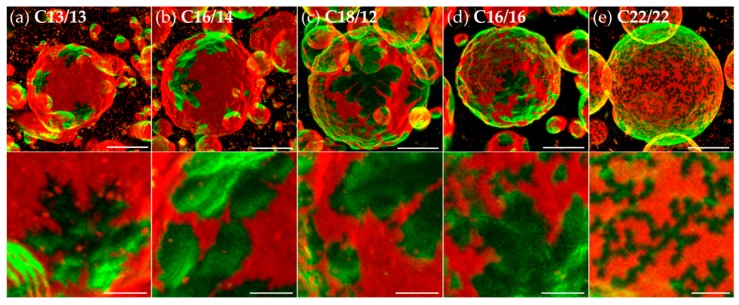
Compact six-fold symmetry stars and patches formed with branched alkyl chains. Confocal 3D projections of GUVs from DPPC and different bolapolyphiles ((**a**) **C13/13** {28}; (**b**) **C16/14** {32}; (**c**) **C18/12** {32}; (**d**) **C16/16** {34} and (**e**) **C22/22** {46}) prepared at 1:10 molar ratio. Images have been sorted from left to right according to increasing lateral chain volume. The autofluorescence of the bolapolyphiles is displayed in green and the fluorescence of the Rh-DPPE counterstain is shown in red. The upper row of panels gives a view of a complete GUV hemisphere (scale bar = 20 µm) for each bolapolyphile. The lower row shows a close-up view of the domain morphology in each image (scale bar = 5 µm).

**Figure 3:**
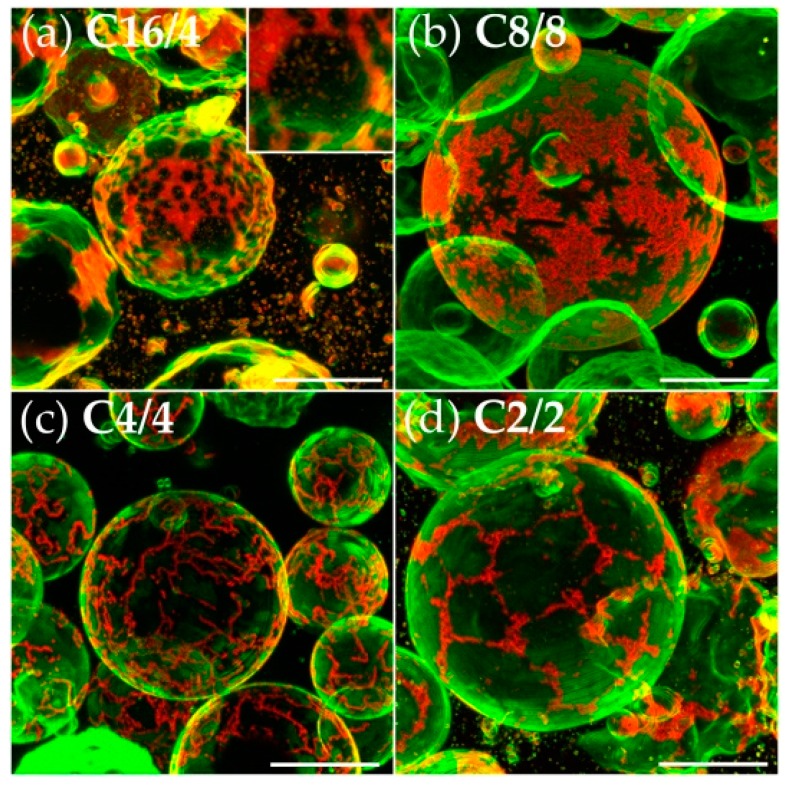
Non-symmetrically branched or short alkyl chains. Confocal 3D projections of GUVs from DPPC and different bolapolyphiles ((**a**) **C16/4** {22}; (**b**) **C8/8** {18}; (**c**) **C4/4** {10} and (**d**) **C2/2** {6}) prepared at a molar ratio of 1:10. The inset in panel (**a**) shows a magnified view of one of the hexagon-shaped domains. Scale bar = 20 µm.

**Figure 4:**
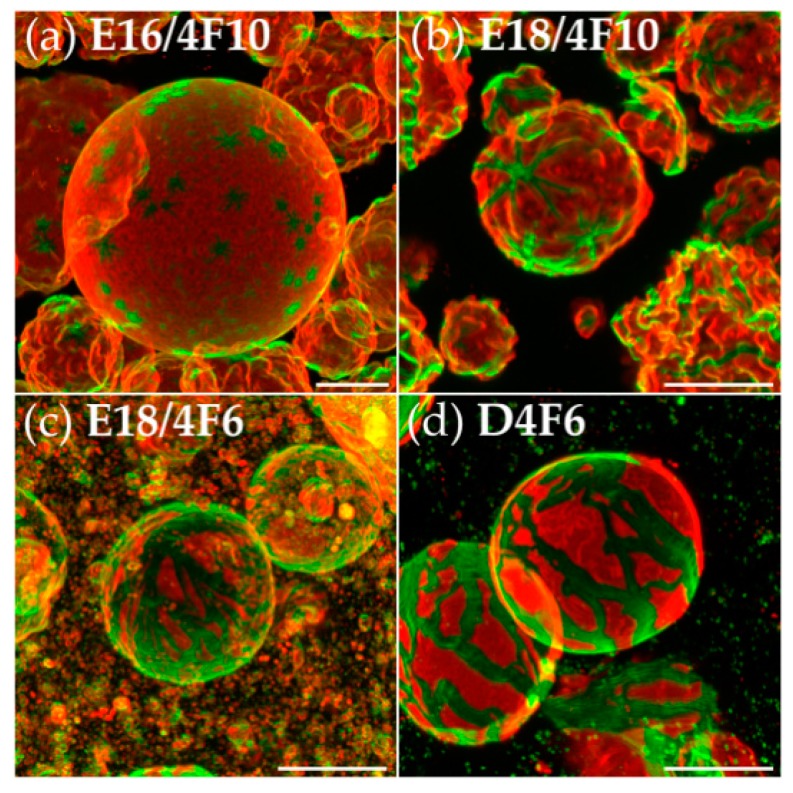
Semiperfluorination of one or both lateral chains. Confocal 3D projections of GUVs from DPPC and different bolapolyphiles ((**a**) **E16/4F10**; (**b**) **E18/4F10**; (**c**) **E18/4F6**; (**d**) **D4F6**) prepared at a molar ratio of 1:10. Scale bar = 20 µm.

**Figure 5:**
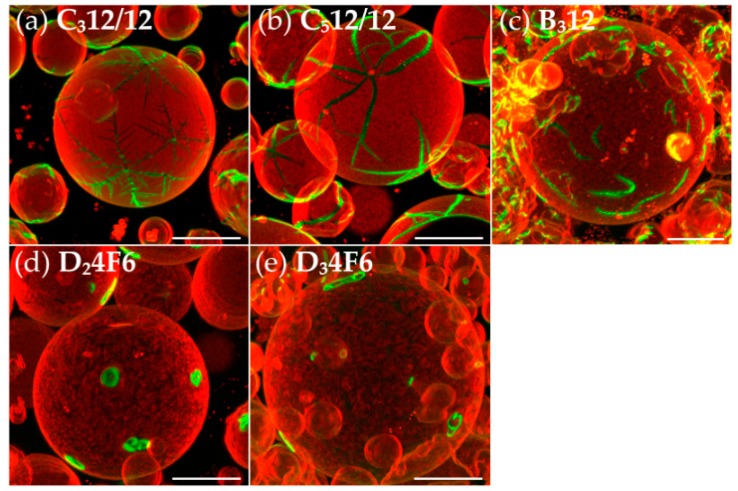
Headgroup extensions by an oligo(ethylene oxide) (EO) moiety in between the OPE core and glycerol. Confocal 3D projections of GUVs from DPPC and different bolapolyphiles ((**a**) **C_3_12/12** {26}, (**b**) **C_5_12/12** {26}, (**c**) **B_3_12** {12}, (**d**) **D_2_4F6**, (**e**) **D_3_4F6**) prepared at a molar ratio of 1:10. A confocal image of GUVs containing **B_3_12** {12} has previously been published in [12]. Scale bar = 20 µm.

Lateral structure formation on the supramolecular scale was observed by confocal fluorescence microscopy (Figure 1, Figure 2, Figure 3, Figure 4 and Figure 5). The bolapolyphiles, depicted in green, were visualized directly, exploiting the intrinsic fluorescence of their π-conjugated OPE cores. A small amount of fluorescent lipidic dye (Rh-DPPE) was added to each bolapolyphile:DPPC mixture, serving as a red counterstain. Rh-DPPE is a head-labeled lipid with fully saturated chains that partitions into *l_d_* phases as well as into DPPC-rich gel-like domains of lower order, but is excluded from DPPC-rich gel phases of high order, as well as from cholesterol-containing *l_o_* phases [24,25,26]. Differential packing of Rh-DPPE into some, but not all gel phases in two-lipid-component systems is not surprising, because the presence of a minor component is known to reduce the order of a DPPC gel phase, removing the lipid tilt, without yet completely fluidizing it [26].

### 3.2. Effects of Variation of the Lipophilic Lateral Chains

We had previously examined the bolapolyphiles **B12** and **B18**, which contain unbranched chains, and had observed the formation of dendritic, bolapolyphile-enriched, Rh-DPPE-depleted star-shaped domains with a clear six-fold symmetry. The OPE cores form π-π-stacked rows, whereas the lateral alkyl chains pack favorably with the acyl chains of the DPPC lipid. The hexagonal symmetry apparently stems from the hexagonal packing of the DPPC in the solid-ordered gel phase, with rows of π-π stacked bolapolyphiles pervading the hexagonally packed DPPC, possibly in a honeycomb configuration [12]. The strong depletion of Rh-DPPE from the star-shaped bolapolyphile-enriched domains corroborates a highly-ordered, dense, hexagonal DPPC packing within the domains. Comparing **B12** to **C12/12** and **B18** to **C18/18**, the length of an unbranched chain in **B12** almost matches the length of one of the branched chains in **C12/12**. The same applies to **B18** and **C18/18**.

**C12/12** and **C18/18** exhibit the same type of six-fold symmetric, dendritic, star-shaped domains as observed with **B12** and **B18** (Figure 1), supporting a fundamentally similar packing behavior of the branched and unbranched analogs. Moreover, an increase in the relative area of the star-shaped (green) domains is observed in the order **B12** < **B18** ≈ **C12/12** < **C18/18**. This trend is explained by the increasing cross-sectional areas of the unpolar parts of the molecule.

All the bolapolyphiles with simple glycerol headgroups have a small cross-section of the hydrophilic headgroup compared to the hydrophobic parts of the molecule (which includes the rod-like-core and the two lateral chains). Hence, the pure bolapolyphiles do not form lamellar phases in bulk and no lyotropic phases could be observed due to the poor solubility in water [11], although they do form a lamellar phase when mixed with phospholipids with larger head groups. In bulk, because of the small head group, saddle-splay curvature develops and a bicontinuous cubic phase is formed for an alkyl chain length of *n* = 10 (see Figure 2c of part 1 [17]), whereas a lamellar phase is observed only after expansion of the headgroups by incorporation of additional EO units (see Table 5 of part 1 [17]). Thus the thermotropic bulk behavior of the bolapolyphiles is affected by headgroup expansion in a similar way as achieved by the coordination of water molecules to the headgroups in the lyotropic liquid-crystalline [27,28]. In the lyotropic lamellar phases of mixtures of bolapolyphiles with the phospholipid DPPC, the small headgroup size is compensated by the larger phosphatidylcholine headgroups in the surrounding lipid matrix. A similar behavior is known for cholesterol (and functionally related sterols) in biological membranes. Cholesterol constitutes a rigid amphiphile with a hydroxy-group as a small headgroup. Like the bolapolyphiles, cholesterol requires neighboring phospholipid headgroups to shield its hydrophobic core from the water and allow the formation of a lamellar phase (umbrella model [29]).

In parallel with the increase in the fraction of DPPC, the molar fraction of bolapolyphile in the domain decreases. This trend is confirmed in the green channel, where the star-shaped domains show a strongly contrasting green fluorescence for **B12** {12}, less contrast for **B18** {18} and for **C12/12** {26} and no clear contrast relative to the surrounding area for **C18/18** {38} (Figure 1, lower panels). For all four bolapolyphiles, the red marker Rh-DPPE is strongly depleted from the highly ordered star-shaped domains, providing the strong contrast in the two-color overlay images.

GUVs from pure DPPC at room temperature have a facetted, corrugated and lacerated appearance [8,12], because the gel-phase DPPC bilayer cannot adopt the necessary membrane curvature. The bolapolyphile-containing GUVs appear more rounded (albeit not uniform) and are completely devoid of hole defects, indicating that the bilayer is fluid or contains only small patches of gel-phase domains. The three-dimensional, ridge-like profile of the six arms of the green-colored stars in Figure 1d (**C18/18**) suggests that each arm and each branch of an arm consists of a gel phase domain with a particular tilt orientation that is incompatible with the tilt orientation of a branch extending from another arm. This incompatibility of tilts probably promotes the star-shaped instead of space-filling growth.

In an earlier study, Dreier et al. [30] used polarized microscopy to investigate the orientational textures of “leaf”-shaped gel phase DPPC-rich domains, emanating from a central defect in planar DPPC/DOPC bilayers. These orientational textures arise due to different tilt orientations of the DPPC acyl chains. The jump angle at the defect lines between “leaves” was found to be near 60°. Dreier et al. also highlighted that when the stiffness of the hexagonal lipid packing is high, areas with uniform orientation separated by line defects are favored over a continuous change in the orientation of tilting. The star-shaped domains formed by DPPC with **B12**, **B18**, **C12/12** and **C18/18** fit into this concept. The strict separation between the branches suggests a strongly ordered hexagonal packing with high stiffness. It is conceivable that this long-range stiff hexagonal packing in the mixed bolapolyphile-DPPC phase is enabled by the C_12_ chains of **B12** and **C12/12** chains crystallizing alongside the DPPC acyl chains, yielding an H-shaped configuration for **C12/12** (Figure 3b, part 1 [17]). The longer C_18_ chains of B18 and C18/18 slightly exceed the lipophilic membrane height and therefore may fold into turns and crystallize partially.

We also examined further bolapolyphiles (**C*m/m’***) with symmetrically branched (*m = m’*) as well as non-symmetrically branched lateral chains (*m ≠ m’*). The six-fold symmetric star shape of the bolapolyphile-enriched, strongly Rh-DPPE-depleted domains is again a striking feature (Figure 2). As seen from left to right in the panels of Figure 2, there is an evolution from stars with a still slightly dendritic appearance (Figure 2a, **C13/13** {28}) to bulky, polygonal stars (Figure 2b–d, **C16/14** {32}, **C18/12** {32}, **C16/16** {34}). This transition is accompanied by the appearance of an increasing number of small, interconnected round or polygonal domains. Ultimately, only the small interconnected domains are observed (Figure 2, **C22/22** {46}).

Different tilt orientations may prevent the small domains (Figure 2c–e) from coalescing into large domains in the same manner as the arms of a star remain separated (Figure 2a–d). Moreover, the increase in volume of the hydrophobic lateral chains on the molecular level may be responsible for the increase in the area fraction of the green domains on the macroscopic level (Figure 2, from left to right), because a larger number of DPPC headgroups is needed to shield bolapolyphiles with larger hydrophobic chain volumes.

It is so far unknown why two different series of morphologies, namely the increase in the area of the dendritic stars as seen in Figure 1 and the transition from stars to more bulky domains as seen in Figure 2, evolve. However, it is clear that there are at least two, probably concurring mechanisms by which an increase in hydrophobic volume can be compensated. The DPPC:bolapolyphile ratio within the domains may increase and lipid tilt may be reduced. Both mechanisms could have different consequences, depending on chain volume and matching of bolapolyphile lateral chains and DPPC chains. Not only very long chains (**C22/22** {46}, Figure 2e) but also a particularly large difference in the length of the branches in the branched compounds **C*m/m’*** (**C16/4** {22}, Figure 3a) result in bulky domains. The spatial resolution of the confocal microscopy does not allow a distinction between polygonal or round shapes in the case of the small, approximately 1 µm-sized domains in Figure 2c–e and Figure 3a. For the larger domains observed with C16/4 in Figure 3a, hexagon-shaped domains are discernable, which support the hypothesis of hexagonal packing.

Finally, we considered bolapolyphiles with significantly shorter lateral chains (**C8/8**, **C4/4**, **C2/2**, Figure 3b–d). All GUVs became rounded, indicating that the bilayer is fluid or contains only small patches of gel-phase domains. With **C4/4** and **C2/2**, strongly Rh-DPPE enriched, bolapolyphile-depleted red-fluorescent streaks pervaded the bolapolyphile-enriched green-fluorescent areas that cover the majority of the GUV surface.

With their short lateral chains, **C4/4** and **C2/2** more closely mimic sterols, such as cholesterol, that form *l_o_* phases with DPPC [31]. Fluorescently labeled phospholipids, such as Rh-DPPE, are generally excluded from these *l_o_* phases [32], explaining the observation that Rh-DPPE is clearly relegated to the DPPC-enriched streaks. There appears to be a trend from **C8/8** {18} over **C4/4** {10} to **C2/2** {6}: The star-shaped green domains (Figure 3b) increase in size and coalesce, this time not because more DPPC is needed to shield particularly bulky lateral chains but due to a better compatibility of the bolapolyphile with DPPC, presumably resulting in an *l_o_* phase with a large area fraction. On a side note, in the thermotropic bulk studies [17] (part 1), an amphiphilic instead of triphilic assembly was observed for **C2/2** and **C4/4** that have short lateral chains. Likewise, in bilayers, the short lateral chains also cause the bolapolyphiles to lose one philicity. When the lateral chains are too short to crystallize alongside the DPPC acyl chains, the driving force for the formation of rigid, hexagonal bolapolyphile:DPPC domains is lost.

### 3.3. Dendritic Growth under Non-Equilibrium Conditions

The GUVs need not be at thermodynamic equilibrium. The polymorphism of dendritic domains (Figure 1) versus more bulky domains (Figure 2), as well as the formation of a few large domains versus many small domains (Figure 2c–e) may be of kinetic origin. Dendritic growth is known to occur under non-equilibrium conditions, when undercooling is paired with the saturation of a melt [33,34]. The morphology of the dendrites is controlled by the chemical nature and structure of the involved substances and crystal growth anisotropy [35,36,37]. Dendritic growth also depends on the undercooling temperature, the latent heat and the rate of heat transport in the system.

Dendritic growth was previously observed in supported bilayers, but not in the free-standing bilayers of GUVs, leading to the conclusion that nucleation depended on the support [38]. To our knowledge, this system is the first one in which dendritic growth is observed in free-standing lipid bilayers [39]. Dendritic growth has been more extensively studied in inorganic systems, for example during the solidification of water and metals. Water forms a variety of six-fold symmetric snowflakes, including dendritic six-fold symmetry stars and hexagonal solid plates [40]. While the polymorphism of snow flake shapes is well appreciated, the routes leading to different types of crystals constitute areas of research. The intricate snowflake shape is determined by the course of the growth conditions that the crystal experiences. In nature, no two snowflakes appear alike, but it has been recently shown that crystals experiencing the same course of growth conditions in the laboratory do acquire similar shapes [40]. Six-fold symmetric morphologies as in Figure 1 were also observed in α-Fe_2_O_3_ crystals and were referred to as “micro-pine” and “micro-snowflake” [36].

Since each GUV forms a closed system with negligible lipid exchange between GUVs during cooling, and since diffusion of the bilayer components is restricted to the two-dimensional plane, diffusion may be the rate-limiting step in crystallization, promoting the dendritic growth of bolapolyphile-DPPC domains.

### 3.4. Semiperfluorination of the Lateral Chains

Semiperfluorination of one of the lateral chains bestows tetraphilicity on the bolapolyphile. As described in part 1 [17], in bulk, i.e., in the absence of water, semiperfluorination substantially stabilizes the honeycomb-like mesophase, because the fluorinated segments (R_F_) are able to interact among themselves and segregate away from the non-fluorinated segments (R_H_). In membrane bilayers, the circumstances are significantly different, because the sandwich geometry, consisting of a hydrophilic, a hydrophobic and a hydrophilic layer, is imposed on the bolapolyphile assembly, leaving no possibility for complete segregation of R_F_. Conceding to this geometrical constraint, the elongated semiperfluorinated C_14_ chains in **E16/4F10** and **E18/4F10** may pack alongside the OPE core and the extended DPPC acyl chains, accounting for the preservation of the star-shaped domains observed with these bolapolyphiles (Figure 4a,b).

The interactions among the semiperfluorinated segments are expected to further stabilize the row-like assembly of bolapolyphiles, but some decoupling between the semiperfluorinated chains and the DPPC patches is expected in comparison to the coupling between alkyl chains and DPPC patches. Softer coupling between the bolapolyphile rows and the hexagonal DPPC patches is expected to reduce the long-range order. Since the C_14_ semiperfluorinated chain in **E16/4F10** and **E18/4F10** still falls within the intermediate range of alkyl chain lengths, suitable for packing with the DPPC acyl chains (C_12_ as in **B12** to C_18_ as in **B18** are suitable), stars are still observed. In contrast, the semiperfluorinated C_10_ chain in **E18/4F6** and **D4F6** appears to be too short, causing further decoupling of the bolapolyphile packing from the hexagonal packing preference of the DPPC. It results in the less ordered, albeit still branched, river-delta-like morphology seen in Figure 4c,d. In line with these observations, the substitution of both C_12_ saturated chains in **B_3_12** by shorter and thicker semiperfluorinated chains (yielding **D_3_4F6**), destabilizes the bulk bolapolyphile crystal, as indicated by a lower melting point (107 °C for **D_3_4F6** compared to 131 °C for **B_3_12** (see Tables 4 and 5 of part 1 [17]).

### 3.5. Oligo(Ethylene Oxide) Expanded Bolapolyphile Headgroups

In the bulk thermotropic phase, all bolapolyphiles with C_12_ or (CH_2_)_4_C_6_F_13_ lateral chains and headgroups expanded by EO groups in between OPE core and glycerol headgroups (**C*_x_*12/12** and D*_x_*4F6 series) exhibit a columnar hexagonal, honeycomb-like mesophase, where the polar columns become enlarged with increasing headgroup size and the OPE packing becomes less ordered (see Tables 3 and 4 and Figure 10 of part 1 [17]). For the largest headgroup (**C_5_12/12**), segregation of polar headgroups and hydrophobic moieties is still retained, but hydrophobic OPE cores and hydrophobic lateral chains mix, resulting in an amphiphilic instead of triphilic assembly.

In the lyotropic lipid bilayers, an additional constraint needs to be considered, namely the shielding of the hydrophobic bolapolyphile moieties from water. However, bolapolyphiles with expanded headgroups (*x* > 0) are less reliant on DPPC headgroups for shielding the hydrophobic core from the water than bolapolyphiles with small headgroups (*x* = 0), causing a gradual decoupling of bolapolyphile packing and domain shape from the hexagonal DPPC lattice with increasing relative headgroup size. While **C_3_12/12** with an expanded headgroup of *x* = 3 and {24} carbons in the lateral chains still shows a clear six-fold symmetric star-shaped domain morphology with straight arms (Figure 5a), a further increase in headgroup size (**C_5_12/12** with *x* = 5) or, alternatively, a decrease in lateral chain volume (**B_3_12** with only {12} carbons in the lateral chains) causes the arms of the star to veer from their straight paths and bend into boomerang shapes (Figure 5b,c). Semiperfluorination of the lateral chains in bolapolyphiles with expanded headgroups (**D_2_4F6**, **D_3_4F6**) further contributes to decoupling of the bolapolyphile packing and DPPC packing, allowing for the domains to further curl into loops (Figure 5d,e). In all cases of expanded headgroups (non-fluorinated lateral chains as well as semifluorinated lateral chains, Figure 5a–e), the bolapolyphile-enriched domains cover only a low area fraction of the GUV surface. This indicates that a low amount of DPPC is present in those bolapolyphile-enriched domains, as expected when the bolapolyphiles are less dependent on DPPC headgroups for the shielding of their hydrophobic cores from the water.

## 4. Conclusions

Simulations have predicted that cholesterol locally disrupts DPPC gel phase packing, reducing hexagonal DPPC clusters to smaller sizes [41]. Bolapolyphiles share the feature of the small headgroup(s) with cholesterol and also pack in between hexagonal DPPC clusters, but they do not abolish the long-range orientational order of the DPPC gel phase. We propose that the polyphilic nature of the compounds, namely the fact that the lateral chains in the bolapolyphiles differ in philicity from the rigid core, but share the philicity of the DPPC chains, leads to an ordered, hexagonal, tilted molecular arrangement, which causes the remarkable macroscopic six-fold symmetric domain morphology. To our knowledge, this domain morphology has not been observed with any other synthetic or natural compounds in free-standing lipid bilayers. The recently proposed model of bolapolyphile-DPPC packing within these dendritic star-shaped domains, which was based on x-ray scattering, NMR spectroscopy and physicochemical data [11,12,13], is supported and refined by the present structure–function study.

**Figure 6:**
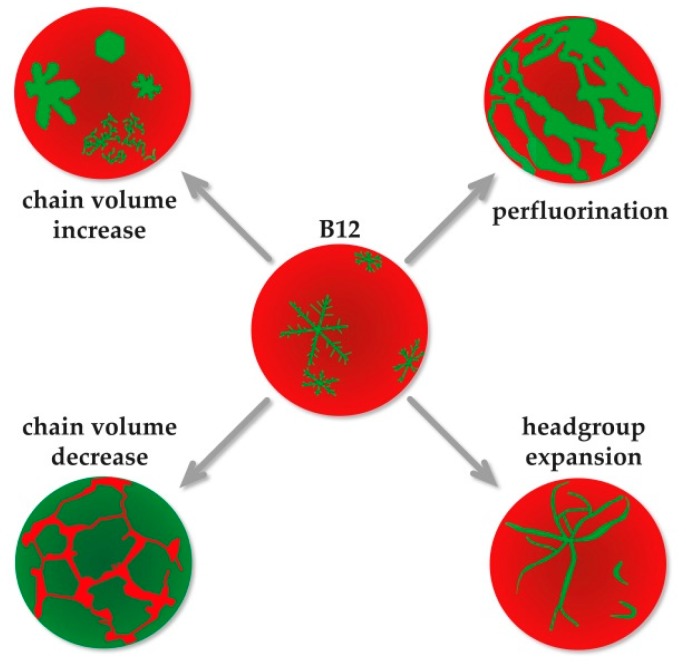
Effect of changes in molecular structure on domain morphologies. Starting from the bolapolyphile **B12**, which yields clear, six-fold symmetric stars with a high bolapolyphile and low DPPC content, this scheme summarizes the morphological effects of changing the lateral chain volume, expanding the headgroups or introducing lateral chain semiperfluorination in free-standing bolapolyphile-DPPC bilayers. From these observations, it is hypothesized that an increase in lateral chain volume leads to more bulky domains with an increased DPPC content due to a need for water shielding (upper left). A decrease in lateral chain volume presumably converts polyphiles into amphiphiles that pack with DPPC in a similar manner as cholesterol, sending the red fluorescent lipid to the remaining DPPC-rich domains (lower left). Both alkyl chain semiperfluorination and headgroup expansion apparently lead to a decoupling of DPPC and bolapolyphile packing, causing a loss of long-range order, which leads to domain curving (upper and lower right). Bolapolyphile headgroup expansion reduces the need for DPPC for water shielding, resulting in domains that cover a small area fraction (lower right).

As summarized in Figure 6, the relative sizes of the flexible lateral chains and headgroups, as well as the chemical nature of the lateral chains, govern the preservation or gradual loss of the six-fold symmetric, star-shaped domain morphology. By considering the four main paths of alterations, the observed modifications of the domain phenotype and their structural implications (Figure 6), compounds may be selected for future detailed structural characterization by spectroscopic and physicochemical techniques.
